# Comorbid Bipolar Disorder and Migraine: From Mechanisms to Treatment

**DOI:** 10.3389/fpsyt.2020.560138

**Published:** 2021-01-11

**Authors:** Jinfeng Duan, Rongmei Yang, Wenwen Lu, Lingfei Zhao, Shaohua Hu, Chenxia Hu

**Affiliations:** ^1^Key Laboratory of Mental Disorder's Management of Zhejiang Province, Department of Psychiatry, School of Medicine, First Affiliated Hospital, Zhejiang University, Hangzhou, China; ^2^Department of Psychogeriatrics, Hangzhou Seventh People's Hospital, Hangzhou, China; ^3^Department of Traditional Chinese Medicine, School of Medicine, First Affiliated Hospital, Zhejiang University, Hangzhou, China; ^4^Key Laboratory of Kidney Disease Prevention and Control Technology, School of Medicine, First Affiliated Hospital, Zhejiang University, Hangzhou, China; ^5^State Key Laboratory for Diagnosis and Treatment of Infectious Diseases, Collaborative Innovation Center for Diagnosis and Treatment of Infectious Diseases, School of Medicine, First Affiliated Hospital, Zhejiang University, Hangzhou, China

**Keywords:** bipolar disorder, migraine, mechanism, treatment, genetics, inflammation, neurotransmitter, mitochondrial dysfunction

## Abstract

Bipolar disorder (BD) is a severe psychiatric disorder characterized by recurrent episodes of manic/hypomanic or depressive symptoms and euthymic periods, with some patients suffering a gradual deterioration of illness and consequent cognitive deficits during the late stage. Migraine is a disease generally without abnormal medical examinations, neurological examinations or laboratory studies, and the diagnosis is made based on the retrospective demonstration of headache features and groupings of disease-associated symptoms. The epidemiology of comorbid BD and migraine is high and it is obligatory to find effective treatments to improve the prognosis. Recent investigations demonstrated that the close relationship between BD and migraine significantly increased the rapid cycling rates of both BD and migraine in patients. Although the detailed mechanism is complex and largely unclear in comorbid BD and migrain, genetic factors, neurotransmitters, altered signaling pathways, disturbances of inflammatory cytokines, and mitochondrial dysfunction are risk factors of BD and migraine. Particularly these two diseases share some overlapping mechanisms according to previous studies. To this end, we call for further investigations of the potential mechanisms, and more efforts are underway to improve the treatment of people with comorbid BD and migraine. In this review, we provide an overview of the potential mechanisms in patients with BD or migraine and we further discuss the treatment strategies for comorbid BD and migraine and it is obligatory to find effective treatments to improve the prognosis. This work will provide insights for us to know more about the mechanisms of comorbid BD and migraine, provides new therapeutic targets for the treatment and give clinicians some guidance for more appropriate and beneficial treatment.

## Introduction

Bipolar disorder (BD) is a severe psychiatric disorder and characterized by recurrent episodes of manic/hypomanic (namely BD-I/BD-II, respectively) or depressive symptoms and euthymic periods, and some patients experience a gradual deterioration of illness and consequent cognitive deficits (1). Therefore, these 2 types of BD was distinguished according to the severity of manic symptoms. Furthermore, BD-II is not severe enough to cause social or occupational functional impairment or hospitalization; in contrast, manic and even psychotic symptoms are more severe in BD-I, and patients often require hospitalization. The incidence rate of symptomatic depression in patients with BD (BD-I or BD-II) are 3 times more than the incidence rate of mania or hypomania (2). The onset of BD occurs predominantly in adolescence or early adulthood. The lifetime prevalence of BD is appropriately 2.1% globally, and the prevalence of subthreshold forms is ~2.4% (3). With a diagnosis of BD, life expectancy decreases by 9 years on average (4), and the completed suicide rates of men and women with BD are 7.8 and 4.9%, respectively (4, 5). After the development of complications related to BD, such as metabolic and cardiovascular diseases, patients with BD will consequently experience poor quality of life, impaired cognitive function, functional impairments and social impairments.

**Figure 1:**
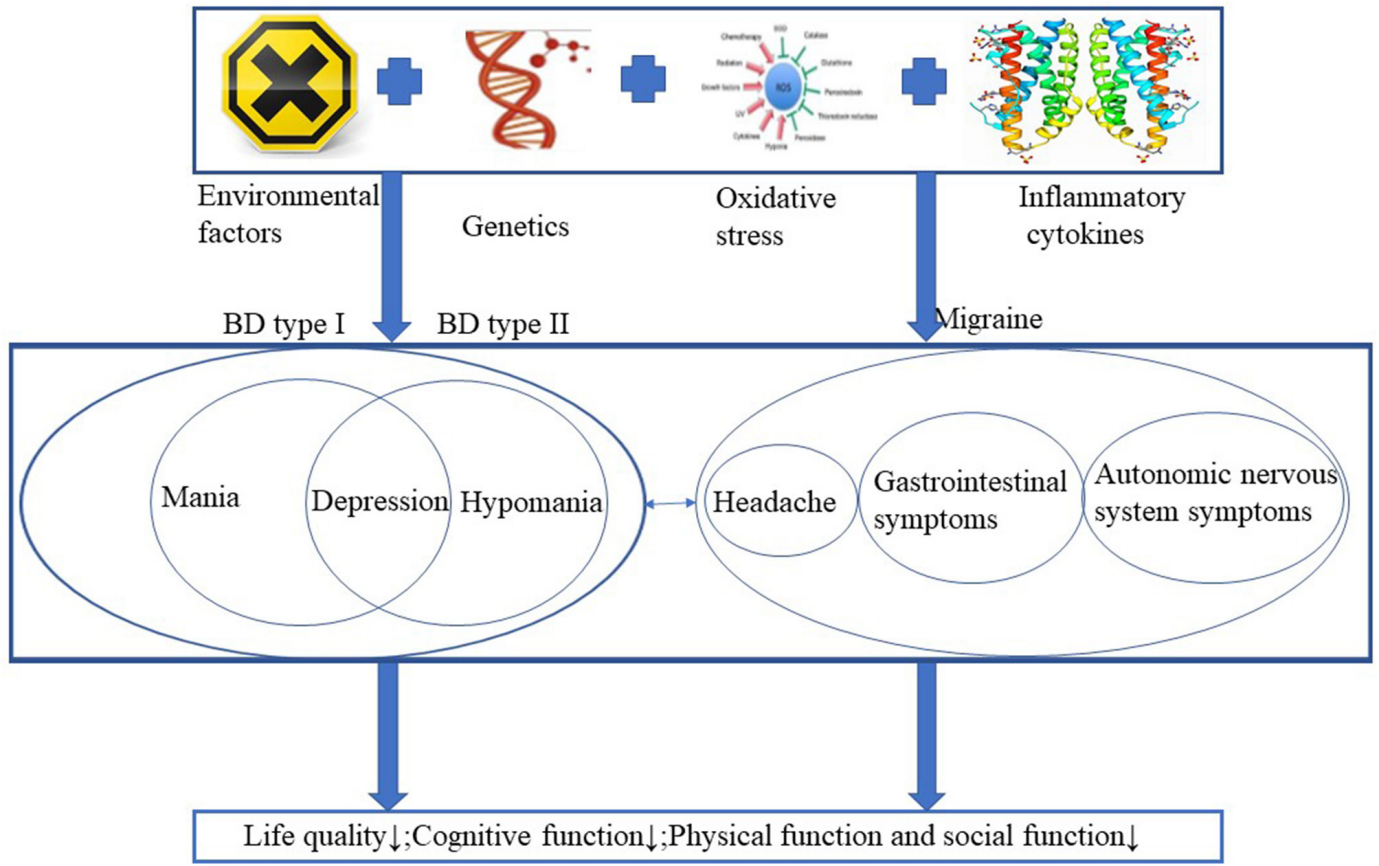
BD and migraine share multiple similar risk factors, including genetic factors, environmental risk factors, oxidative stress and disturbances of inflammatory cytokines. Both diseases lead to decreased quality of life and multiple dysfunctions in humans.

Migraine is a disease without abnormal medical examinations, neurological examinations or laboratory findings, and the diagnosis is made based on retrospective demonstration of headache features and groupings of disease-associated symptoms. Patients with migraine frequently experience episodic attacks, including recurrent headache, gastrointestinal symptoms, and autonomic nervous system symptoms (6). Migraine can also results in decreased quality of life, impaired cognitive function, disturbed brain function and social impairments (7). The prevalence rate of migraine in healthy individuals was 14% with the lifetime prevalence in men was 6%, and 17.6% in women in the United States (8, 9). Psychiatric disorders are common in multiple neurological disorders, migraine is one of the disorders with a high prevalence, and there is a heritable link between BD and migraine (10, 11). Even migraine is not caused by psychiatric illness, and a large proportion of people with migraine are not diagnosed with any comorbid psychiatric disorder. Parental migraine was associated with increased likelihood and a risk factor for offspring BD even in the absence of parental BD, the prevalence of migraine in the BD population may be as high as 39% and rapid cycling as a feature of bipolar disorder and comorbid migraine (10, 12, 13). BD and migraine share multiple similar risk factors, including genetic factors, environmental risk factors, oxidative stress and disturbances of inflammatory cytokines. Both diseases lead to decreased quality of life and multiple dysfunctions in humans (Figure 1). Patients with comorbid BD and migraine have poorer treatment outcomes and increased disability (14). In this review, we provide an overview of the potential mechanisms in patients with BD or migraine and we further discuss the overlapping mechanisms and treatment strategies for comorbid BD and migraine, the potential treatments for patients with comorbid BD and migraine, with the purpose of giving clinicians some guidance for more appropriate and beneficial treatment to the comorbidity.

## Methods

We conducted a systematic search of two major databases: PubMed and Embase from January 1991 to July 2020. The search terms used were “bipolar disorder/s, manic depressive, manic depression, comorbidity, and migraine.” The terms were cross-referenced to yield a comprehensive search. We also conducted manual searches of bibliographies of reviewed articles. We included in this review original research articles of descriptive, controlled and animal studies and several review articles. The search was limited to English language journals.

### Potential Mechanisms of BD

Multiple pathophysiological processes, such as genetic abnormalities, abnormal regulation of neurotransmitters, altered signaling pathways, reduced neurotrophic factors, inflammatory disturbances, mitochondrial dysfunction, cell apoptosis and impaired cell resilience, may be involved in the development of BD. The interaction of these processes leads to abnormal neuronal function which may result in mood instability, disturbed energy metabolism, abnormal biological rhythms and cognition defects (Table 1). Patients with BD present an increased rate of DNA damage compared to that of controls, which is closely related to the severity of BD symptoms (32). The gray matter volume showed by the positron emission tomography (PET) was significantly decreased in the subgenual prefrontal cortex sections from BD patients, which was closely related to a reduced glial cell number and neuronal cell number or size (33, 34). Calcium signaling, particularly through voltage-gated calcium channels, was thought to play vital roles in the pathogenesis and treatment of BD, the expression of the calcium channel Cav1.2 subunit, which is encoded by *CACNA1C* (the most likely BD gene), was also abnormally regulated in the pathogenesis of BD (15, 35). Combined data from genome-wide association studies (GWASs) and gene expression experiments revealed that hormone regulation, second messenger pathways, glutamatergic transmission and histone expression, even the immune system were all involved in the pathogenesis of BD (36, 37). Monoamines and gamma-amino butyric acid (GABA) are important in the pathophysiology of BD (16). In addition, serum brain-derived neurotrophic factor (BDNF) is decreased (17), while neurotrophin-3 (NT-3) (18) is upregulated in patients with BD who are experiencing a manic state or a depressive state. Furthermore, controlling the release of BDNF contributes to the effective treatment of BD, and BDNF has been reported to regulate the cell survival rate in individuals with BD (38).

**Table 1:** Multiple pathophysiological processes participate in the development of BD.

**Author, Date**	**Type**	**Potential mechanism**	**Regulation**	**Result**	**References**
S. Bhat, 2012	Genetics	*CACNA1C*	Abnormally regulated	More robust associations in BD patients than in depression or schizophrenia patients	(15)
Y. Oda, 2012	Neurotransmitters	GABA-inhibitory interneuronal activity	Dysfunction	Deficits in gamma band oscillations in BD patients	(16)
K. Hashimoto, 2004	Neurotrophic factor	Serum brain-derived neurotrophic factor (BDNF)	Downregulated	Stress-induced neuronal damage; impaired neurogenesis in the hippocampus	(17)
J. C. Walz, 2007	Neurotrophic factor	Neurotrophin-3 (NT-3)	Upregulated	Higher in manic and depressed BD patients than in euthymic patients and healthy controls	(18)
Y. K. Kim, 2007	Inflammatory factors	IL-6/IL-4,TNF-α/IL-4, IL-2/IL-4, and IFN-γ/IL-4 ratios	Upregulated	Higher in manic BD patients than in healthy controls	(19)
Y. K. Kim, 2007	Inflammatory factors	IL-4	Downregulated	Lower in BD patients than in healthy controls	(19)
J. S. Rao, 2010	Inflammatory factors	NMDA receptors NR-1 and NR-3A	Downregulated	Lower in BD patients than in healthy controls	(20)
J. S. Rao, 2010	Inflammatory factors	IL-1β, IL-1 receptor, myeloid differentiation factor 88 (MyD88), nuclear factor-kappa B subunits, glial fibrillary acidic protein (GFAP), inducible nitric oxide synthase (iNOS), c-fos and CD11b	Upregulated	Higher in BD patients than in healthy controls	(20)
J. S. Rao, 2010	Inflammatory factors	Tumor necrosis factor-alpha (TNF-α) and nNOS	Not altered	Equivalent in BD patients and healthy controls	(20)
Modabbernia, 2013	Inflammatory factors	TNF-α and its receptor, IL-6 and its receptor, IL-2 receptor, IL-4, IL-10, and IL-1 receptor antagonist (IL-1RA)	Upregulated	Higher in BD patients than in healthy controls	(21)
K. Munkholm, 2013	Inflammatory factors	soluble IL-2 receptor (sIL-2R), TNF-α, soluble tumor necrosis factor receptor type 1 (sTNFR1), sIL-6R and IL-4	Upregulated	Higher in BD patients than in healthy controls	(22)
K. Munkholm, 2013	Inflammatory factors	TNF-α, sTNF-R1, sIL-2R	Upregulated	Higher in manic BD patients than in healthy controls	(23)
K. Munkholm, 2013	Inflammatory factors	sTNF-R1 and TNF-α	Upregulated	Higher in manic BD patients than in euthymic BD patients	(23)
K. Munkholm, 2013	Inflammatory factors	sTNF-R1	Upregulated	Higher in euthymic BD patients than in healthy controls	(23)
Y. M. Bai, 2014	Inflammatory factors	sTNF-R1	Downregulated	Lower in bipolar II patients than in bipolar I patients; lower in depressive BD patients than in manic/hypomanic/euthymic BD patients	(24)
T. Kato, 1993	Mitochondrial dysfunction	pH	Upregulated	Higher in manic BD patients than in euthymic BD patients	(25)
T. Kato, 1993	Mitochondrial dysfunction	pH	Downregulated	Lower in euthymic BD patients than in healthy controls	(25)
X. Sun, 2006	Mitochondrial dysfunction	Complex I, complex IV and complex V	Downregulated	Lower in BD patients than in healthy controls	(26)
N. Buttner, 2007	Mitochondrial dysfunction	DNA fragmentation	Upregulated	Higher in BD patients than in schizophrenic patients or healthy controls	(27)
R. E. Riegel, 2009	Mitochondrial dysfunction	TBARS and superoxide generation	Upregulated	Higher in rats with a mania-like state than in normal rats	(28)
J. F. Wang, 2009	Mitochondrial dysfunction	4-hydroxynonenal	Upregulated	Higher in BD patients than in healthy controls; 4-HNE levels significantly correlated with pH values only in BD patients	(29)
M. Kunz, 2008	Mitochondrial dysfunction	SOD activity	Upregulated	Higher in manic and depressed BD patients than in healthy controls/euthymic BD patients	(30)
M. Kunz, 2008	Mitochondrial dysfunction	TBARS	Upregulated	Higher in euthymic/manic/depressed BD patients than in healthy controls	(30)
M. Yumru, 2009	Mitochondrial dysfunction	The total antioxidant status (TAS), total oxidant status (TOS) and oxidative stress index (OSI)	Upregulated	Higher in BD patients than in healthy controls	(31)
M. Yumru, 2009	Mitochondrial dysfunction	TOS	Upregulated	Higher in BD I patients than in BD II patients	(31)

Patients with BD always exhibit a disturbed balance between pro-inflammatory factors and anti-inflammatory factors. The peripheral levels of inflammatory factors, including the interleukin (IL) family, IFN-gamma (IFN-γ) and tumor necrosis factor-alpha (TNF-α), are upregulated in the manic episode of BD patients compared to those in healthy controls, while the level of IL-4 is downregulated in BD patients compared to that in healthy controls (19). Modabbernia et al. demonstrated significantly elevated levels of TNF-α and its receptor, IL-6 and its receptor, IL-2 receptor, IL-4, IL-10, and IL-1 receptor antagonist (IL-1RA) in patients with BD compared to those in healthy individuals (21, 22). In addition, individuals with BD who are experiencing mania have been shown to express higher levels of TNF-α, sTNF-R1, and sIL-2R than healthy controls, and the levels of sTNF-R1 and TNF-α in manic BD patients are higher than those in patients experiencing euthymia. Furthermore, the expression level of sTNF-R1 is significantly upregulated in patients experiencing euthymia compared to that in healthy controls (23). The expression level of the TNF receptor was more significantly decreased in BD patients experiencing a depressive state than in those experiencing a manic/hypomanic state or euthymic state, and this expression level was also downregulated in BDII patients compared to that in BD I patients (24). Adiponectin is decreased in bipolar depression, and might interfere with the pathophysiological mechanisms of BD and its somatic comorbidities via involvement in metabolic and inflammatory processes (39). Glial inflammatory factors, including glial fibrillary acidic protein, nitric oxide (NO) synthase, c-fos and CD11b, and astroglial inflammatory factors, including myeloid differentiation factor 88, nuclear factor-kappa β (NF-κβ), cyclooxygenase, prostaglandin-E synthase, IL-1β and IL-1 receptor, were significantly upregulated in the postmortem frontal cortex of BD patients compared to those in healthy controls, thus indicating that microglial and astroglial activation are promoted during the pathogenetic process of BD (20). However, another study showed that the levels of the N-methyl-D-aspartate (NMDA)receptors NR-1 and NR-3A were downregulated in BD patients compared to those in healthy controls, and the levels of TNF-α and Neural Nitric Oxide Synthase(nNOS) were not altered in these patients (20).

On the other hand, mitochondrial dysfunction results in the impairment of cell resilience and participates in the initiation and progression of BD (40). BD has also been demonstrated to be associated with impaired respiratory complex function and the induction of cellular degeneration (41). Oxidative stress occurs after an imbalance of redox homeostasis is initiated, accompanied by overexpression of free radicals or deficiencies in the antioxidant response. The pH was increased in cerebral tissue from manic patients compared to that in cerebral tissue from euthymic BD patients, but was decreased in cerebral tissue from euthymic BD patients compared to that in cerebral tissue from healthy controls (25). Moreover, expression of mitochondrial electron transport chain-related genes, such as complex I, complex IV and complex V, were downregulated in the frontal cortex of BD patients compared to that in healthy controls (26). In another postmortem study, Buttner et al. showed that oxidative stress-induced DNA fragmentation was enhanced in non-GABAergic neurons in the anterior cingulate cortex of BD patients compared to that of schizophrenic patients or healthy controls (27). Ouabain significantly increased superoxide generation, lipid peroxidation and thiobarbituric acid reactive substances (TBARS) to induce the generation of rats in a mania-like state (28). Moreover, Wang et al. found that the level of 4-hydroxynonenal (4-HNE, a major product of lipid peroxidation) was significantly increased in postmortem anterior cingulate brain sections from BD patients compared to the healthy controls, and 4-HNE levels were significantly correlated with pH values in BD patients (29). However, superoxide dismutase (SOD) activity was found to be upregulated only in patients experiencing acute phases of BD, such as manic episode or depressive episode, but not altered in patients experiencing an euthymic state of BD or healthy controls. In addition, the TBARS level was upregulated in BD patients at all stages compared to that in healthy controls (30). The expression level of 3-nitrotyrosine is upregulated during the early and late stages of BD; in contrast, the expression levels of glutathione reductase and glutathione S-transferase (GST) are significantly upregulated in patients during the late stage of BD compared to those in patients during the early stage of BD. Consequently, cumulative oxidative stress promoted the release of these antioxidant enzymes to reduce further oxidative stress-induced damage during the progression of BD. The total antioxidant status, total oxidant status and oxidative stress index are significantly upregulated in BD patients compared to those in healthy controls. Furthermore, the fact that BD-I is more severe than BD-II may be attributed to the higher total oxidant status in BD-I patients than in BD-II patients (31).

### Potential Mechanisms of Migraine

Patients with migraine are more susceptible to headaches and associated clinical symptoms when they are experiencing a hyperexcitable brain state, and they perceive painful emotions and experiences. However, the potential mechanisms underlying the pathophysiology of migraine should be further investigated to shed light on the treatment of this complicated disease (Table 2). The dysfunction of descending pain modulatory circuits, which leads to a loss of pain inhibition and hyperexcitability in nociceptive areas of the brain (53). Dopamine receptors are involved in the determination of migraine trait, and migraine patients with dopaminergic symptoms are characterized by a full-blown, more disabling migraine (54). After scanning five patients who did not receive any migraine prophylaxis by PET, Afridi et al. found that there was a significant activation of the dorsolateral pons during spontaneous migraine attacks and concluded that migraine was a kind of subcortical disorder (55). More recently, other imaging studies have shown that there is also a significant activation of posterior/dorsal thalamic sections in patients with spontaneous migraine (56). Pain signals in the brain are transferred centrally to the trigeminal nucleus caudalis and the trigeminocervical complex, which then sends fibers to the thalamus and the autonomic nuclei, and finally, the thalamic neurons project to the somatosensory cortex and parts of the limbic system. This progress of neural communication is mediated by a number of neuropeptides and neurotransmitters, including monoamines (57). Altered perception of stimuli that is not painful promotes the reflection of pain and activates the feed-forward neurovascular dilator mechanism in the first division of the trigeminal nerve. After stimulation with functional 5-hydroxy tryptamine in the trigeminal nerve endings, the release of calcitonin gene-related peptide was decreased, and a mild level of vasoconstriction could be triggered by stimulating receptors on meningeal blood vessels and the trigeminal nucleus caudalis, thus resulting in decreased central neuronal signaling (57). Monoclonal antibodies to Calcitonin Gene-related Peptide (CGRP) or its receptors, have proven efficacy on migraine prevention, in both episodic and chronic migraine. Kainate and NMDA receptor antagonists, pituitary adenylate cyclase-activating polypeptide(PACAP) type 1 receptor (PAC1) agonists and Kynurenic Acid(KYNA) analogs are still in preclinical phase for the treatment of migraine (58).

**Table 2:** The potential underlying mechanism during the pathophysiological process of migraine.

**Author, Date**	**Type**	**Potential mechanism**	**Regulation**	**Result**	**References**
R. A. Ophoff, 1996	Genetics	CACNA1A	Polymorphic variations (a (CA)n-repeat (D19S1150), a (CAG)n-repeat in the 3'-UTR) and different types of deleterious mutations	FHM	(42)
E. Garza-Lopez, 2012	Genetics	G protein-dependent modulation of mutations W1684R and V1696I	Affects the apparent dissociation and reassociation rates of the Gβγ dimer	G protein-Ca(2+) channel affinity may be altered in FHM type I	(43)
M. De Fusco, 2003	Genetics	ATP1A2	Loss of function of a single allele of ATP1A2	FHM type II	(44)
M. Dichgans, 2005	Genetics	SCN1A	A heterozygous missense mutation (Gln1489Lys) in the neuronal voltage-gated sodium channel gene	FHM type III	(45)
R. Burstein, 2010	Inflammatory factors	Blood oxygenation level-dependent (BOLD) signals	Stronger BOLD responses	Migraine attack with extracephalic allodynia compared to the corresponding responses	(46)
F. Perini, 2005	Inflammatory factors	IL-10, TNF-α, and IL-1β	Upregulated	Higher in patients during attacks than outside of attacks	(47)
F. Perini, 2005	Inflammatory factors	Serum levels of IL-10 and TNF-α	Upregulated/downregulated	Higher in migraine patients soon after headache onset and lower over time	(47)
P. P. Bruno, 2007	Inflammatory factors	Chemokines	Upregulated	Stimulate the activation of trigeminal nerves	(48)
P. P. Bruno, 2007	Mitochondrial dysfunction	Nitric oxide (NO)	Upregulated	Induces inflammation in migraine patients	(48)
J. Olesen, 2010	Mitochondrial dysfunction	NO	Upregulated	Causes headache in normal volunteers and a delayed headache in migraine patients	(49)
C. Bernecker, 2011	Mitochondrial dysfunction	4-hydroxy-2-nonenal (HNE)	Upregulated	Higher in female migraine patients than in healthy controls; HNE is significantly correlated with the nitric oxide pathway and with insulin and lipid metabolism	(50)
M. Neri, 2015	Mitochondrial dysfunction	NO	Upregulated	Higher in migraine patients with aura during attacks	(51)
M. Neri, 2015	Mitochondrial dysfunction	Thiobarbituric acid reactive substances (TBARS)	Upregulated	Higher in migraine patients than in healthy controls	(51)
I. Ciancarelli, 2004	Mitochondrial dysfunction	Urinary levels of NO and TBARS	Upregulated	Higher in migraine patients than in healthy controls	(52)

Migraine may also be induced by brainstem dysfunction, which initiates perimeningeal vasodilatation and neurogenic inflammation. As the potential mechanisms of migraine are extraordinarily complex, inflammation is a salient factor in the pathophysiology of migraine. The levels of blood oxygenation level-dependent (BOLD) signals are significantly elevated in migraine attacks with extracephalic allodynia compared to those in the corresponding responses (46). In addition, levels of the well-known pro-inflammatory cytokines, including IL-10, TNF-α, and IL-1β were significantly upregulated during acute migraine attacks compared to those outside of acute attacks, while the levels of IL-10 and TNF-α were increased in migraine patients soon after headache onset but decreased over time (47). The release of chemokines was increased in migraine patients and stimulated the activation of trigeminal nerves (48). Oxidative stress, such as NO, serves as a critical factor in the pathophysiology of migraine, and an elevated level of NO-induced inflammation has been reported in migraine patients (48). Moreover, increased expression of NO induced headache in normal volunteers and delayed headache in migraine patients (49). 4-Hydroxy-2-nonenal levels are upregulated in female migraine patients compared to those in healthy controls and have been shown to be significantly correlated with the NO pathway, insulin metabolism and lipid metabolism (50). NO and TBARS levels were upregulated in patients with migraine compared to those in healthy individuals (51, 52). In recent years, studies have shown that imbalances between oxidative stress and the antioxidative response participate in the pathophysiology of migraine, as the levels of oxidative stress-related genes and enzymes are elevated, whereas the levels of antioxidant genes and enzymes are diminished during the progression of migraine.

### The Overlapping Mechanisms of BD and Migraine

In recent years, the potential mechanisms of comorbid BD and migraine have been widely investigated, and future research focused on genetic factors, brain imaging, mitochondrial dysfunction and inflammatory factors may further elucidate the underlying mechanisms. Epidemiological and clinical studies have showed a high degree of comorbidity between BD and migraine, they two may have multifactorial polygenic etiology and share common pathophysiology (59). Since there is a strong bidirectional association between migraine and BD, revealing the potential overlapping neurobiological mechanisms of these two diseases could promote the development of novel treatments. Parental migraine has been demonstrated to serve as a risk factor for offspring BD, even in patients without parental BD, and there seems to be a common genetic factor between BD and migraine. However, BD is more closely related to comorbid migraine than parental migraine, and this elevated comorbidity may be attributed to nongenetic factors (60). Both of BD and migraine are closely associated with abnormalities in serotonergic pathways, dopaminergic pathways, and glutaminergic systems. Central serotonergic activity is reduced during the depressive and euthymic phases of BD (61), while the serotonin level is low in migraine patients between attacks and is upregulated after the initiation of a migraine attack (62). As dopamine is a well-recognized factor in migraine pathophysiology, dopamine receptor antagonists, as prochlorperazine, chlorpromazine, metoclopramide, and promethazine are first-line agents in the emergency room setting for migraine (63). The levels of glutamate were significantly upregulated in the anterior cingulate cortex and downregulated in the hippocampus in BD patients compared to those in controls (64). Glutamate from platelets was released and amino acids was increased in migraine patients with aura and without aura, although this increase was more significant in migraine patients with aura. Platelet glutamate uptake, assessed by 3H-glutamate intake, was increased in migraine patients with aura, but it was reduced in migraine patients without aura compared to that in healthy controls (65). The blood levels of leptin and adiponectin of the migraineurs are associated with disease pathogenesis of migraine (66).

Calcium channels Cav1, Cav2, and Cav3 are the targets of mutations and polymorphisms that alter their function and regulation can lead to neuropsychiatric diseases, including migraine and BD (67). Genome-wide linkage studies in BD and migraine patients proved that there were overlapping areas of linkage on chromosomes. *CACNA1A* and *CACNA1C*, which are voltage-dependent calcium channels, have recently been proven to play critical roles in FHM and BD. Furthermore, there is a locus on chromosome 20p11 with overlapping elevated logarithm of odds scores for both migraine and BD, and the locus harbors a well-known gene, namely, *SLC24A3*, which encodes a potassium-dependent sodium/calcium exchanger for maintaining calcium homeostasis in nervous tissue (68). Although another study found that there was no relationship between rs10994336 in *ANK3* during the progression of BD and rs1006737 in *CACNA1C* during the progression of migraine, this result may be attributed to the small sample size (69). Oedegaard et al. used GWAS to compare BD patients without headache and BD patients with migraine and found that there were nine single nucleotide polymorphism (SNP) (Chr13:41192397-41388566) values in chromosome 13q14.1 in BD patients with migraine, and genetic variants of the *KIAA0564* gene region may predispose to migraine headaches in patients with BD. The strongest relationship was reported for several single nucleotide polymorphisms in a 317-kb region, whereas rs9566845 and rs9566867 remained the most prominent genetic variants in this study (70). Dopamine pathway genes, including LIM homeobox transcription factor 1, alpha (LMX1A) and neuregulin 1 (NRG1), are associated with cognitive performance in BD patients, the rs35753505 SNP was associated with increased performance, while the rs11809911 SNP in LMX1A was associated with reduced IQ and memory (71).

Furthermore, it has been reported that the pathophysiologic mechanisms of BD and migraine can be attributed to chronic inflammation, a disturbance of the balance between oxidative stress and the antioxidative stress response, and the regulation of nitrosative stress (72, 73). Panx1 channels and Connexins 43 hemichannels appears to be involved in inflammation and has been documented in migraine and BD (74). Furthermore, targeting the inflammatory pathways may decrease the cooccurrence of BD and migraine; thus, the elucidation of the related inflammatory pathways may offer new pharmacological strategies (75, 76). In addition, these inflammatory cytokines may serve as biomarkers for the prediction of outcomes. These pathological mechanisms may induce cross-sensitization between BD and migraine, thus shifting the illnesses to a more severe form, and patients with both diseases respond poorly to pharmacotherapies, with relapsing acute mood episodes, cognitive dysfunction and functional deficiency, and decreased life expectancy.

### Current Treatments of BD and Migraine

#### Current Treatments of BD

Patients with BD theoretically experience interspersed euthymia and relapsing mood episodes of depressive and manic status (77), while actual BD symptoms are more complex, and the patients experience mixed mood states and potential cognitive impairments (78). As BD is termed as a dynamic and fluctuating disease, the control of this lifelong disease is challenging. During the early stages, patients with BD respond favorably to psychiatric and psychosocial therapy and exhibit less cognitive dysfunction and fewer functional impairments, but they exhibit accelerated rapid cycling of episodes, severe brain structural abnormalities, a higher prevalence of other comorbid diseases, and many more abnormal peripheral biomarkers after the illness progresses (79).

Mood stabilizers, including the univalent ion lithium, valproate, lamotrigine, and carbamazepine, serve as the cornerstones of therapy, and individual atypical antipsychotic medications are emerged as common choices to control acute manic/hypomanic and acute depression in order to maintain the treatment during the remission phase (80). Although patients with BD sometimes experience a normal mood state, they may also experience persistent mood instability during this period (81). Moreover, the relapse rate is high even in patients receiving combination treatment. Depression or mania will recur in 37% of patients within 1 year and in 60% of patients within 2 years (82). Mood episodes, especially mania, have been demonstrated to be related to cell death of neuronal cells and glial cells, which is induced by apoptotic inflammatory cytokines (83). Thus, it is urgent for investigators to develop new drugs to eliminate these inflammatory factors targeting at these disorders; lithium and valproate exert protective effects through their immunomodulatory properties in BD patients (75). Traditional mood stabilizers for BD significantly downregulate the levels of phospholipase A_2_ (PLA_2_) and cyclooxygenase (COX), which directly participate in the regulation of immunity and inflammatory cytokine release (84), and other drugs, including nonsteroidal anti-inflammatory drugs (NSAIDs) and glucocorticoids, also exert anti-inflammatory activity to ameliorate BD symptoms (85). In addition to anti-inflammatory function, mood stabilizers (lithium and valproate) upregulate SOD activity and glutathione activity and downregulate the generation of oxidative stress and mitochondrial dysfunction in BD (86). Long-term oral administration of lamotrigine or olanzapine significantly upregulated GST-M1 expression levels and GST activity in rat cerebral cortical cells, which suggested that GST-M1 may serve as an important drug target for the treatment of BD (87). Patients receiving behavioral and cognitive-behavioral therapies are free from the adverse effects of chemical drugs; in addition, such therapies make it easier for the patients to control their own disease by modifying distorted thinking, increasing their motivation to participate in pleasurable activities and improving their problem-solving abilities, and these therapies can be easily added to integrated therapy. The combination of pharmacotherapy, behavioral and cognitive-behavioral therapies improves medication adherence, delaying disease recurrence and improving the maintenance of treatment gains in patients with BD (88).

#### Current Treatments of Migraine

The symptoms of patients with migraine can be influenced by environmental triggers, including bad eating habits, sleep disorders, stressful stimulation, emotional status, hormone fluctuations in women, and weather changes (57). The current main treatment for moderate-to-severe migraine is serotonin receptor agonists, namely, triptans (89). After treatment with a triptan and another serotonergic agent, migraine patients may experience serotonin syndrome (90), and clinicians must pay attention to control the side effects of these agents. Several preventive medications, including anti-depressive drugs, antiepileptic drugs, hypotensive drugs, dietary supplements, herbal medicines, and botulinum toxin, are widely used as effective preventive treatments for clinical migraine symptoms (91). The comorbidity of migraine with evening chronotype BD patients is higher than compared with non-evening types of BD, and exogenous melatonin supplementation plays the potential prophylactic role in patients with episodic migraine, even there is no conclusive evidence comparing the efficacy of exogenous melatonin supplementation for migraine prophylaxis to the other FDA-approved pharmacotherapy (92, 93). Doctors can choose a targeted medication according to the patient's general health state, current comorbidities, additional drugs and personal preferences. In addition to medications, other strategies, including lifestyle changes, self-management techniques, and relaxation, can be used to control the symptoms of migraine in patients (94).

#### Potential Treatments for Patients With Comorbid BD and Migraine

There are still no optimal alternative treatments for patients suffering from comorbid BD and migraine, but several pharmacological treatments, such as valproate, lithium, lamotrigine, quetiapine and topiramate, are widely used to prevent the onset of both migraine attacks and acute manic or depressive episodes in patients with BD (95, 96). Patients with comorbid BD and migraine were younger and more educated and had a family history of either disease, but they had fewer hospitalizations for psychiatric disorders. The initial symptom for such patients is depression rather than hypomania or mania, and they are prescribed fewer mood stabilizers but more atypical antidepressants by their doctors. Improper antidepressant treatment used and analgesic drug abuse in cormobid patients is not rare, which leads to the reduction threshold of pain in cormobid BD with migraine patients, and then, may further interfer the patients' clinical medication (97). BD patients with migraine experience missed diagnosis and a lack of effective treatments; 27.9% of these patients receive antimigraine medications (triptans) (98). A study demonstrated that 1.8% of the total study population received specific treatments for migraine, and 0.45% of the population received a mood-stabilizing agent for BD; among these patients, only 843 in 4,640,219 individuals received both types of medications. Moreover, there was a strong positive association between treatment with migraine medications and treatment with mood-stabilizing agents (99). In recent years, comorbidity with BD has garnered increased attention, particularly in the psychiatric literature. Some treatments may exert adverse effects on the comorbid condition of BD and migraine, and that a tricyclic antidepressant may induce mania and aggravate the progression of disease (100). For patients with comorbid BD and migraine, mood stabilizer maybe the useful treatment, include lithium, anticonvulsant drugs and atypical antipsychotics. In the presence of mixed features of mood episodes, patients with comorbid BD and migraine should be better prescribed with sodium valproate, lamotrigine priority rather than lithium (101). Some specific psychoactive medications such as cognitive behavioral therapy and social rhythm therapy also have effects on the treatment of both BD and migraine (102).To this end, additional clinical and preclinical experimental studies should expand the investigation of new therapies for treating both BD and migraine.Deep transcranial magnetic stimulation (dTMS) (one of the new physical stimulation techniques used for the treatment of different neuropathologies) was also explored as a possible treatment for BD and migraine and may have beneficial neurocognitive effects by targeting on the left dorsolateral prefrontal cortex (DLPFC) (103). Electrical neuromodulation approaches as vagus nerve stimulation (VNS) is one of the treatment to migraine and may cause changes in leptin and associated mediators of immunometabolic signaling, with higher at baseline level of IL-10 and elevated IL-1b (104, 105). Improving the clinicians' recognition and diagnosis of comorbid BD with migraine accurately is of great importance.

## Conclusions

There are several limitations in this review. Firstly, included studies in this review are not systematic and may be intrinsically susceptible to bias, we cannot guarantee the quality and pertinence of these studies. Secondly, animal models of BD and migraine are scarce for further investigation of the mechanisms and treatments. Thirdly, we are not able to get a conclusion about the treatments between BD and migraine since the diagnosis varies according to different researches. Lastly, although the overlapping biological mechanisms seem to be genetic factors, abnormal neurotransmitters, inflammation, and mitochondrial dysfunction, and these factors cooperate to initiate and accelerate the progression of BD and migraine. It is hard to find a specific strategy to improve the therapeutic effects of the comorbidity according to current studies since the underling mechanisms are very complex and migraine symptoms may exacerbate BD symptoms and interfere with BD management. In recent years, inflammation and mitochondrial dysfunction have attracted interest from multiple investigators, and they have been shown to exert important effects on the pathogenesis of BD and migraine. By this way, an improved understanding of the overlapping mechanisms during the pathogenesis of comorbid BD and migraine will significantly improve the diagnosis and treatment then improve the therapeutic effects for patients with comorbid BD and migraine. In our opinion, although preventive medications, including mood-stabilizing agents and serotonergic agents, are widely used in patients with BD and migraine, respectively, additional effort is needed. To improve the prognosis of patients with comorbid BD and migraine, the identification of more effective and less toxic drugs and the improvement of poor compliance are essential. Safe and efficacious neuromodulatory approaches offer the prospect of treatment on comorbid BD with migraine in the future, as clinical researches on TMS or VNS to treat comorbid BD and migraine are expected to show effective therapeutic and cognitive improvement, as well as long-term follow-up studies about the changes in inflammatory factors, leptin and adipokines before and after treatment. Also experiments exploring the mechanisms of comorbid BD and migraine are necessary. In the near future, we are looking forward to find an effective treatment targeting on the intersectional mechanism for alleviating illness of patients with comorbid BD and migraine.

## Author Contributions

CH conceived the original idea. JD, RY, WL, and LZ wrote the article. SH revised this manuscript, which all authors reviewed and approved for publication.

## Conflict of Interest

The authors declare that the research was conducted in the absence of any commercial or financial relationships that could be construed as a potential conflict of interest.

## Abbreviations

BD: bipolar disorder
GABA: gamma-amino butyric acid
GDNF: glial cell-derived neurotrophic factor
NT-3: neurotrophin-3
BDNF: brain-derived neurotrophic factor
IL: interleukin
IFN-γ: interferon-gamma
TNF-α: tumor necrosis factor-alpha
NO: nitric oxide
NF-κβ: nuclear factor-kappa β subunits
sTNFR1: soluble TNF-α receptor-1
sIL-6R: soluble IL-6 receptor
IL-1RA: IL-1 receptor antagonist
sIL-2R: soluble IL-2 receptor
TBARS: thiobarbituric acid reactive substances
GST: glutathione S-transferase
PET: positron emission tomography
FHM: familial hemiplegic migraine
PLA_2_: phospholipase A_2_
COX: cyclooxygenase
NSAIDs: nonsteroidal anti-inflammatory drugs
AA: arachidonic acid
TMS: transcranial magnetic stimulation
DLPFC: left dorsolateral prefrontal cortex.

## References

[1] Kapczinski F Dias VV Kauer-Sant'Anna M Frey BN Grassi-Oliveira R Colom F Clinical implications of a staging model for bipolar disorders. Expert Rev Neurother. (2009) 9:957–66. 10.1586/ern.09.31 19589046

[2] Joffe RT MacQueen GM Marriott M Trevor Young L A prospective, longitudinal study of percentage of time spent ill in patients with bipolar I or bipolar II disorders. Bipolar Disord. (2004) 6:62–6. 10.1046/j.1399-5618.2003.00091.x 14996142

[3] Merikangas KR Akiskal HS Angst J Greenberg PE Hirschfeld RM Petukhova M. Lifetime and 12-month prevalence of bipolar spectrum disorder in the National Comorbidity Survey replication. Arch Gen Psychiatry. (2007) 64:543–52. 10.1001/archpsyc.64.5.543 17485606 PMC1931566

[4] Crump C Sundquist K Winkleby MA Sundquist J. Comorbidities and mortality in bipolar disorder: a Swedish national cohort study. JAMA Psychiatry. (2013) 70:931–9. 10.1001/jamapsychiatry.2013.1394 23863861

[5] Nordentoft M Mortensen PB Pedersen CB. Absolute risk of suicide after first hospital contact in mental disorder. Arch Gen Psychiatry. (2011) 68:1058–64. 10.1001/archgenpsychiatry.2011.113 21969462

[6] Haut SR Bigal ME Lipton RB. Chronic disorders with episodic manifestations: focus on epilepsy and migraine. Lancet Neurol. (2006) 5:148–57. 10.1016/S1474-4422(06)70348-9 16426991 PMC1457022

[7] Lofland JH Frick KD. Workplace absenteeism and aspects of access to health care for individuals with migraine headache. Headache. (2006) 46:563–76. 10.1111/j.1526-4610.2006.00404.x 16643549

[8] Dilsaver SC Benazzi F Oedegaard KJ Fasmer OB Akiskal KK Akiskal HS. Migraine headache in affectively ill latino adults of mexican american origin is associated with bipolarity. Prim Care Comp J Clin Psychiatry. (2009) 11:302–6. 10.4088/PCC.08m00728 20098521 PMC2805565

[9] Lipton RB Stewart WF Simon D Medical consultation for migraine: results from the American Migraine Study. Headache. (1998) 38:87–96. 10.1046/j.1526-4610.1998.3802087.x 9529763

[10] Sinha A Shariq A Said K Sharma A Jeffrey Newport D Salloum IM. Medical comorbidities in bipolar disorder. Curr Psychiatry Rep. (2018) 20:36. 10.1007/s11920-018-0897-8 29732528

[11] Hesdorffer DC. Comorbidity between neurological illness and psychiatric disorders. CNS Spectr. (2016) 21:230–8. 10.1017/S1092852915000929 26898322

[12] McIntyre RS Konarski JZ Wilkins K Bouffard B Soczynska JK Kennedy SH. The prevalence and impact of migraine headache in bipolar disorder: results from the Canadian Community Health Survey. Headache. (2006) 46:973–82. 10.1111/j.1526-4610.2006.00469.x 16732843

[13] Gordon-Smith K Forty L Chan C Knott S Jones I Craddock N. Rapid cycling as a feature of bipolar disorder and comorbid migraine. J Affect Disord. (2015) 175:320–4. 10.1016/j.jad.2015.01.024 25661398 PMC4366040

[14] Jeyagurunathan A Abdin E Vaingankar JA Chua BY Shafie S Chang SHS. Prevalence and comorbidity of migraine headache: results from the Singapore Mental Health Study 2016. Soc Psychiatry Psychiatr Epidemiol. (2020) 55:33–43. 10.1007/s00127-019-01755-1 31456029

[15] Bhat S Dao DT Terrillion CE Arad M Smith RJ Soldatov NM. CACNA1C (Cav1.2) in the pathophysiology of psychiatric disease. Prog Neurobiol. (2012) 99:1–14. 10.1016/j.pneurobio.2012.06.001 22705413 PMC3459072

[16] Oda Y Onitsuka T Tsuchimoto R Hirano S Oribe N Ueno T. Gamma band neural synchronization deficits for auditory steady state responses in bipolar disorder patients. PLoS ONE. (2012) 7:e39955. 10.1371/journal.pone.0039955 22792199 PMC3390322

[17] Hashimoto K Shimizu E Iyo M. Critical role of brain-derived neurotrophic factor in mood disorders. Brain Res Brain Res Rev. (2004) 45:104–14. 10.1016/j.brainresrev.2004.02.003 15145621

[18] Walz JC Andreazza AC Frey BN Cacilhas AA Cereser KM Cunha AB. Serum neurotrophin-3 is increased during manic and depressive episodes in bipolar disorder. Neurosci Lett. (2007) 415:87–9. 10.1016/j.neulet.2007.01.002 17234344

[19] Kim YK Jung HG Myint AM Kim H Park SH. Imbalance between pro-inflammatory and anti-inflammatory cytokines in bipolar disorder. J Affect Disord. (2007) 104:91–5. 10.1016/j.jad.2007.02.018 17434599

[20] Rao JS Harry GJ Rapoport SI Kim HW. Increased excitotoxicity and neuroinflammatory markers in postmortem frontal cortex from bipolar disorder patients. Mol Psychiatry. (2010) 15:384–92. 10.1038/mp.2009.47 19488045 PMC2844920

[21] Modabbernia A Taslimi S Brietzke E Ashrafi M. Cytokine alterations in bipolar disorder: a meta-analysis of 30 studies. Biol Psychiatry. (2013) 74:15–25. 10.1016/j.biopsych.2013.01.007 23419545

[22] Munkholm K Brauner JV Kessing LV Vinberg M. Cytokines in bipolar disorder vs. healthy control subjects: a systematic review and meta-analysis. J Psychiatr Res. (2013) 47:1119–33. 10.1016/j.jpsychires.2013.05.018 23768870

[23] Munkholm K Vinberg M Vedel Kessing L. Cytokines in bipolar disorder: a systematic review and meta-analysis. J Affect Disord. (2013) 144:16–27. 10.1016/j.jad.2012.06.010 22749156

[24] Bai YM Su TP Tsai SJ Wen-Fei C Li CT Pei-Chi T. Comparison of inflammatory cytokine levels among type I/type II and manic/hypomanic/euthymic/depressive states of bipolar disorder. J Affect Disord. (2014) 166:187–92. 10.1016/j.jad.2014.05.009 25012430

[25] Kato T Takahashi S Shioiri T Inubushi T. Alterations in brain phosphorous metabolism in bipolar disorder detected by *in vivo* 31P and 7Li magnetic resonance spectroscopy. J Affect Disord. (1993) 27:53–9. 10.1016/0165-0327(93)90097-4 8432961

[26] Sun X Wang JF Tseng M Young LT. Downregulation in components of the mitochondrial electron transport chain in the postmortem frontal cortex of subjects with bipolar disorder. J Psychiatry Neurosci. (2006) 31:189–96. 16699605 PMC1449880

[27] Buttner N Bhattacharyya S Walsh J Benes FM. DNA fragmentation is increased in non-GABAergic neurons in bipolar disorder but not in schizophrenia. Schizophr Res. (2007) 93:33–41. 10.1016/j.schres.2007.01.030 17442540 PMC1991302

[28] Riegel RE Valvassori SS Elias G Reus GZ Steckert AV de Souza B. Animal model of mania induced by ouabain: evidence of oxidative stress in submitochondrial particles of the rat brain. Neurochem Int. (2009) 55:491–5. 10.1016/j.neuint.2009.05.003 19447152

[29] Wang JF Shao L Sun X Young LT. Increased oxidative stress in the anterior cingulate cortex of subjects with bipolar disorder and schizophrenia. Bipolar Disord. (2009) 11:523–9. 10.1111/j.1399-5618.2009.00717.x 19624391

[30] Kunz M Gama CS Andreazza AC Salvador M Cereser KM Gomes FA. Elevated serum superoxide dismutase and thiobarbituric acid reactive substances in different phases of bipolar disorder and in schizophrenia. Prog Neuropsychopharmacol Biol Psychiatry. (2008) 32:1677–81. 10.1016/j.pnpbp.2008.07.001 18657586

[31] Yumru M Savas HA Kalenderoglu A Bulut M Celik H Erel O. Oxidative imbalance in bipolar disorder subtypes: a comparative study. Prog Neuropsychopharmacol Biol Psychiatry. (2009) 33:1070–4. 10.1016/j.pnpbp.2009.06.005 19527764

[32] Andreazza AC Frey BN Erdtmann B Salvador M Rombaldi F Santin A. DNA damage in bipolar disorder. Psychiatry Res. (2007) 153:27–32. 10.1016/j.psychres.2006.03.025 17582509

[33] Drevets WC Price JL Simpson JR Jr Todd RD Reich T. Subgenual prefrontal cortex abnormalities in mood disorders. Nature. (1997) 386:824–7. 10.1038/386824a0 9126739

[34] Ongur D Drevets WC Price JL. Glial reduction in the subgenual prefrontal cortex in mood disorders. Proc Natl Acad Sci USA. (1998) 95:13290–5. 10.1073/pnas.95.22.13290 9789081 PMC23786

[35] Berridge MJ. Calcium signalling and psychiatric disease: bipolar disorder and schizophrenia. Cell Tissue Res. (2014) 357:477–92. 10.1007/s00441-014-1806-z 24577622

[36] Nurnberger JI Jr Koller DL Jung J Edenberg HJ Foroud T Guella I. Identification of pathways for bipolar disorder: a meta-analysis. JAMA Psychiatry. (2014) 71:657–64. 10.1001/jamapsychiatry.2014.176 24718920 PMC4523227

[37] Network T O'Dushlaine C Rossin L Lee PH Duncan L Parikshak NN Psychiatric genome-wide association study analyses implicate neuronal, immune and histone pathways. Nat Neurosci. (2015) 18:199–209. 10.1038/nn.3922 25599223 PMC4378867

[38] Kapczinski F Frey BN Kauer-Sant'Anna M Grassi-Oliveira R. Brain-derived neurotrophic factor and neuroplasticity in bipolar disorder. Expert Rev Neurother. (2008) 8:1101–13. 10.1586/14737175.8.7.1101 18590480

[39] Platzer M Fellendorf FT Bengesser SA Birner A Dalkner N Hamm C. Adiponectin is decreased in bipolar depression. World J Biol Psychiatry. (2019) 20:813–20. 10.1080/15622975.2018.1500033 30047831

[40] Kato T Kato N. Mitochondrial dysfunction in bipolar disorder. Bipolar Disord. (2000) 2:180–90. 10.1034/j.1399-5618.2000.020305.x 11256685

[41] Calabrese V Scapagnini G Giuffrida Stella AM Bates TE Clark JB. Mitochondrial involvement in brain function and dysfunction: relevance to aging, neurodegenerative disorders and longevity. Neurochem Res. (2001) 26:739–64. 10.1023/A:1010955807739 11519733

[42] Ophoff RA Terwindt GM Vergouwe MN van Eijk R Oefner PJ Hoffman SM. Familial hemiplegic migraine and episodic ataxia type-2 are caused by mutations in the Ca2+ channel gene CACNL1A4. Cell. (1996) 87:543–52. 10.1016/S0092-8674(00)81373-2 8898206

[43] Garza-Lopez E Sandoval A Gonzalez-Ramirez R Gandini MA Van den Maagdenberg A De Waard M. Familial hemiplegic migraine type 1 mutations W1684R and V1696I alter G protein-mediated regulation of Ca(V)2.1 voltage-gated calcium channels. Biochim Biophys Acta. (2012) 1822:1238–46. 10.1016/j.bbadis.2012.04.008 22549042

[44] De Fusco M Marconi R Silvestri L Atorino L Rampoldi L Morgante L. Haploinsufficiency of ATP1A2 encoding the Na+/K+ pump alpha2 subunit associated with familial hemiplegic migraine type 2. Nat Genet. (2003) 33:192–6. 10.1038/ng1081 12539047

[45] Dichgans M Freilinger T Eckstein G Babini E Lorenz-Depiereux B Biskup S. Mutation in the neuronal voltage-gated sodium channel SCN1A in familial hemiplegic migraine. Lancet. (2005) 366:371–7. 10.1016/S0140-6736(05)66786-4 16054936

[46] Burstein R Jakubowski M Garcia-Nicas E Kainz V Bajwa Z Hargreaves R. Thalamic sensitization transforms localized pain into widespread allodynia. Ann Neurol. (2010) 68:81–91. 10.1002/ana.21994 20582997 PMC2930514

[47] Perini F D'Andrea G Galloni E Pignatelli F Billo G Alba S. Plasma cytokine levels in migraineurs and controls. Headache. (2005) 45:926–31. 10.1111/j.1526-4610.2005.05135.x 15985111

[48] Bruno PP Carpino F Carpino G Zicari A. An overview on immune system and migraine. Eur Rev Med Pharmacol Sci. (2007) 11:245–8. 17876959

[49] Olesen J. Nitric oxide-related drug targets in headache. Neurotherapeutics. (2010) 7:183–90. 10.1016/j.nurt.2010.03.006 20430317 PMC5084099

[50] Bernecker C Ragginer C Fauler G Horejsi R Moller R Zelzer S. Oxidative stress is associated with migraine and migraine-related metabolic risk in females. Eur J Neurol. (2011) 18:1233–9. 10.1111/j.1468-1331.2011.03414.x 21518147

[51] Neri M Frustaci A Milic M Valdiglesias V Fini M Bonassi S. A meta-analysis of biomarkers related to oxidative stress and nitric oxide pathway in migraine. Cephalalgia. (2015) 35:931–7. 10.1177/0333102414564888 25573894

[52] Ciancarelli I Tozzi-Ciancarelli MG Di Massimo C Marini C Carolei A. Flunarizine effects on oxidative stress in migraine patients. Cephalalgia. (2004) 24:528–32. 10.1111/j.1468-2982.2003.00705.x 15196294

[53] Burch R. Headache currents commentary. Headache. (2013) 53:E1. 10.1111/head.12021 23278836

[54] Barbanti P Aurilia C Egeo G Fofi L Guadagni F Ferroni P. Dopaminergic symptoms in migraine: a cross-sectional study on 1148 consecutive headache center-based patients. Cephalalgia. (2020) 40:1168–76. 10.1177/0333102420929023 32484361

[55] Afridi SK Giffin NJ Kaube H Friston KJ Ward NS Frackowiak RS. A positron emission tomographic study in spontaneous migraine. Arch Neurol. (2005) 62:1270–5. 10.1001/archneur.62.8.1270 16087768

[56] Noseda R Burstein R. Migraine pathophysiology: anatomy of the trigeminovascular pathway and associated neurological symptoms, CSD, sensitization and modulation of pain. Pain. (2013) 154:44–53 (Suppl. 1). 10.1016/j.pain.2013.07.021 23891892 PMC3858400

[57] Ward TN. Migraine diagnosis and pathophysiology. Continuum. (2012) 18:753–63. 10.1212/01.CON.0000418640.07405.31 22868539

[58] Ferroni P Barbanti P. Biomarkers in migraine headache: prognostic and therapeutic implications. Curr Med Chem. (2019) 26:6188–90. 10.2174/092986732634191112142448 31849286

[59] Di Marco O Di Mauro S. Bipolar disorder and chronic migraine: an open problem. Neurol Sci. (2018) 39:85–6. 10.1007/s10072-018-3356-8 29904879

[60] Sucksdorff D Brown AS Chudal R Heinimaa M Suominen A Sourander A. Parental and comorbid migraine in individuals with bipolar disorder: a nationwide register study. J Affect Disord. (2016) 206:109–14. 10.1016/j.jad.2016.07.034 27472412 PMC5077692

[61] Mahmood T Silverstone T. Serotonin and bipolar disorder. J Affect Disord. (2001) 66:1–11. 10.1016/S0165-0327(00)00226-3 11532527

[62] Deen M Christensen CE Hougaard A Hansen HD Knudsen GM Ashina M. Serotonergic mechanisms in the migraine brain - a systematic review. Cephalalgia. (2017) 37:251–64. 10.1177/0333102416640501 27013238

[63] Ong JJY De Felice M. Correction to: migraine treatment: current acute medications and their potential mechanisms of action. Neurotherapeutics. (2018) 15:525–6. 10.1007/s13311-017-0599-7 29313274 PMC5935634

[64] Ehrlich A Schubert F Pehrs C Gallinat J. Alterations of cerebral glutamate in the euthymic state of patients with bipolar disorder. Psychiatry Res. (2015) 233:73–80. 10.1016/j.pscychresns.2015.05.010 26050195

[65] Vaccaro M Riva C Tremolizzo L Longoni M Aliprandi A Agostoni E. Platelet glutamate uptake and release in migraine with and without aura. Cephalalgia. (2007) 27:35–40. 10.1111/j.1468-2982.2006.01234.x 17212681

[66] Abbasi M Noori-Zadeh A Seidkhani-Nahal A Kaffashian M Bakhtiyari S Panahi S. Leptin, adiponectin, and resistin blood adipokine levels in migraineurs: systematic reviews and meta-analyses. Cephalalgia. (2019) 39:1010–21. 10.1177/0333102418807182 30798617

[67] Nanou E Catterall WA. Calcium channels, synaptic plasticity, neuropsychiatric disease. Neuron. (2018) 98:466–81. 10.1016/j.neuron.2018.03.017 29723500

[68] Oedegaard KJ Greenwood TA Lunde A Fasmer OB Akiskal HS Kelsoe JR. A genome-wide linkage study of bipolar disorder and co-morbid migraine: replication of migraine linkage on chromosome 4q24, and suggestion of an overlapping susceptibility region for both disorders on chromosome 20p11. J Affect Disord. (2010) 122:14–26. 10.1016/j.jad.2009.06.014 19819557 PMC5660919

[69] Wober-Bingol C Tropeano M Karwautz A Wagner G Campos-de-Sousa S Zesch HE. No association between bipolar disorder risk polymorphisms in ANK3 and CACNA1C and common migraine. Headache. (2011) 51:796–803. 10.1111/j.1526-4610.2011.01858.x 21395576

[70] Oedegaard KJ Greenwood TA Johansson S Jacobsen KK Halmoy A Fasmer OB. A genome-wide association study of bipolar disorder and comorbid migraine. Genes Brain Behav. (2010) 9:673–80. 10.1111/j.1601-183X.2010.00601.x 20528957 PMC2970709

[71] Rolstad S Palsson E Ekman CJ Eriksson E Sellgren C Landen M. Polymorphisms of dopamine pathway genes NRG1 and LMX1A are associated with cognitive performance in bipolar disorder. Bipolar Disord. (2015) 17:859–68. 10.1111/bdi.12347 26534905

[72] Brown NC Andreazza AC Young LT. An updated meta-analysis of oxidative stress markers in bipolar disorder. Psychiatry Res. (2014) 218:61–8. 10.1016/j.psychres.2014.04.005 24794031

[73] Berk M Kapczinski F Andreazza AC Dean OM Giorlando F Maes M. Pathways underlying neuroprogression in bipolar disorder: focus on inflammation, oxidative stress and neurotrophic factors. Neurosci Biobehav Rev. (2011) 35:804–17. 10.1016/j.neubiorev.2010.10.001 20934453

[74] Sarrouilhe D Dejean C Mesnil M. Connexin43- and pannexin-based channels in neuroinflammation and cerebral neuropathies. Front Mol Neurosci. (2017) 10:320. 10.3389/fnmol.2017.00320 29066951 PMC5641369

[75] Knijff EM Breunis MN Kupka RW de Wit HJ Ruwhof C Akkerhuis GW. An imbalance in the production of IL-1beta and IL-6 by monocytes of bipolar patients: restoration by lithium treatment. Bipolar Disord. (2007) 9:743–53. 10.1111/j.1399-5618.2007.00444.x 17988365

[76] Durham P Papapetropoulos S. Biomarkers associated with migraine and their potential role in migraine management. Headache. (2013) 53:1262–77. 10.1111/head.12174 23848170

[77] Phillips ML Kupfer DJ. Bipolar disorder diagnosis: challenges and future directions. Lancet. (2013) 381:1663–71. 10.1016/S0140-6736(13)60989-7 23663952 PMC5858935

[78] Bourne C Aydemir O Balanza-Martinez V Bora E Brissos S Cavanagh JT. Neuropsychological testing of cognitive impairment in euthymic bipolar disorder: an individual patient data meta-analysis. Acta Psychiatr Scand. (2013) 128:149–62. 10.1111/acps.12133 23617548

[79] Kapczinski F Dias VV Kauer-Sant'Anna M Brietzke E Vazquez GH Vieta E. The potential use of biomarkers as an adjunctive tool for staging bipolar disorder. Prog Neuropsychopharmacol Biol Psychiatry. (2009) 33:1366–71. 10.1016/j.pnpbp.2009.07.027 19666076

[80] Geddes JR Miklowitz DJ. Treatment of bipolar disorder. Lancet. (2013) 381:1672–82. 10.1016/S0140-6736(13)60857-0 23663953 PMC3876031

[81] Broome MR Saunders KE Harrison PJ Marwaha S. Mood instability: significance, definition and measurement. Br J Psychiatry. (2015) 207:283–5. 10.1192/bjp.bp.114.158543 26429679 PMC4589661

[82] Gitlin MJ Swendsen J Heller TL Hammen C Relapse and impairment in bipolar disorder. Am J Psychiatry. (1995) 152:1635–40. 10.1176/ajp.152.11.1635 7485627

[83] McAfoose J Koerner H Baune BT. The effects of TNF deficiency on age-related cognitive performance. Psychoneuroendocrinology. (2009) 34:615–9. 10.1016/j.psyneuen.2008.10.006 19028017

[84] Rao JS Rapoport SI. Mood-stabilizers target the brain arachidonic acid cascade. Curr Mol Pharmacol. (2009) 2:207–14. 10.2174/1874467210902020207 20021459 PMC2825027

[85] Stolk P Souverein PC Wilting I Leufkens HG Klein DF Rapoport SI. Is aspirin useful in patients on lithium? A pharmacoepidemiological study related to bipolar disorder. Prostaglandins Leukot Essent Fatty Acids. (2010) 82:9–14. 10.1016/j.plefa.2009.10.007 19939659 PMC2818404

[86] de Sousa RT Zarate CA Jr Zanetti MV Costa AC Talib LL Gattaz WF. Oxidative stress in early stage bipolar disorder and the association with response to lithium. J Psychiatr Res. (2014) 50:36–41. 10.1016/j.jpsychires.2013.11.011 24332923 PMC4052827

[87] Bakare A Shao L Cui J Young LT Wang JF. Mood stabilizing drugs lamotrigine and olanzapine increase expression and activity of glutathione S-transferase in primary cultured rat cerebral cortical cells. Neurosci Lett. (2009) 455:70–3. 10.1016/j.neulet.2009.03.022 19429109

[88] Lipchik GL Smitherman TA Penzien DB Holroyd KA. Basic principles and techniques of cognitive-behavioral therapies for comorbid psychiatric symptoms among headache patients. Headache. (2006) 46:S119–32. 10.1111/j.1526-4610.2006.00563.x 17034390

[89] Worthington I Pringsheim T Gawel MJ Gladstone J Cooper P Dilli E. Canadian Headache Society Guideline: acute drug therapy for migraine headache. Can J Neurol Sci. (2013) 40:S1–80. 10.1017/S0317167100017819 23968886

[90] Shapiro RE Tepper SJ. The serotonin syndrome, triptans, and the potential for drug-drug interactions. Headache. (2007) 47:266–9. 10.1111/j.1526-4610.2006.00691.x 17300366

[91] Pringsheim T Davenport W Mackie G Worthington I Aube M Christie SN. Canadian Headache Society guideline for migraine prophylaxis. Can J Neurol Sci. (2012) 39:S1−59. 10.1017/S0317167100015109 22683887

[92] Tseng PT Yang CP Su KP Chen TY Wu YC Tu YK. The association between melatonin and episodic migraine: a pilot network meta-analysis of randomized controlled trials to compare the prophylactic effects with exogenous melatonin supplementation and pharmacotherapy. J Pineal Res. (2020) 69:e12663. 10.1111/jpi.12663 32347977

[93] Romo-Nava F Blom TJ Cuellar-Barboza AB Winham SJ Colby CL Nunez NA. Evening chronotype as a discrete clinical subphenotype in bipolar disorder. J Affect Disord. (2020) 266:556–62. 10.1016/j.jad.2020.01.151 32056926

[94] Sorbi MJ Balk Y Kleiboer AM Couturier EG. Follow-up over 20 months confirms gains of online behavioural training in frequent episodic migraine. Cephalalgia. (2017) 37:236–50. 10.1177/0333102416657145 27558500

[95] Karimi N Tavakoli M Charati JY Shamsizade M Single-dose intravenous sodium valproate (Depakine) vs. dexamethasone for the treatment of acute migraine headache: a double-blind randomized clinical trial. Clin Exp Emerg Med. (2017) 4:138–45. 10.15441/ceem.16.199 29026887 PMC5635457

[96] Rej S Yu C Shulman K Herrmann N Fischer HD Fung K Medical comorbidity, acute medical care use in late-life bipolar disorder: a comparison of lithium, valproate, other pharmacotherapies. Gen Hosp Psychiatry. (2015) 37:528–32. 10.1016/j.genhosppsych.2015.07.001 26254672

[97] Oedegaard KJ Riise T Dilsaver SC Lund A Akiskal HS Fasmer OB. A pharmaco-epidemiological study of migraine and antidepressant medications: complete one year data from the Norwegian population. J Affect Disord. (2011) 129:198–204. 10.1016/j.jad.2010.09.009 20889212

[98] Low NC Du Fort GG Cervantes P. Prevalence, clinical correlates, and treatment of migraine in bipolar disorder. Headache. (2003) 43:940–9. 10.1046/j.1526-4610.2003.03184.x 14511270

[99] Oedegaard KJ Dilsaver SC Hundal O Riise T Lund A Akiskal HS. Are migraine and bipolar disorders comorbid phenomena?: findings from a pharmacoepidemiological study using the Norwegian Prescription Database. J Clin Psychopharmacol. (2011) 31:734–9. 10.1097/JCP.0b013e318235f4e9 22020352

[100] Belmaker RH. Bipolar disorder. N Engl J Med. (2004) 351:476–86. 10.1056/NEJMra035354 15282355

[101] Chiossi L Negro A Capi M Lionetto L Martelletti P. Sodium channel antagonists for the treatment of migraine. Exp Opin Pharmacother. (2014) 15:1697–706. 10.1517/14656566.2014.929665 24941134

[102] Leo RJ Singh J. Migraine headache and bipolar disorder comorbidity: a systematic review of the literature and clinical implications. Scand J Pain. (2016) 11:136–45. 10.1016/j.sjpain.2015.12.002 28850455

[103] Tendler A Roth Y Barnea-Ygael N Zangen A. How to use the H1 deep transcranial magnetic stimulation coil for conditions other than depression. J Vis Exp. (2017) 119:e55100. 10.3791/55100 28190035 PMC5352287

[104] Kinfe TM Buchfelder M Chaudhry SR Chakravarthy KV Deer TR Russo M. Leptin and associated mediators of immunometabolic signaling: novel molecular outcome measures for neurostimulation to treat chronic pain. Int J Mol Sci. (2019) 20:4737. 10.3390/ijms20194737 31554241 PMC6802360

[105] Chaudhry SR Lendvai IS Muhammad S Westhofen P Kruppenbacher J Scheef L. Inter-ictal assay of peripheral circulating inflammatory mediators in migraine patients under adjunctive cervical non-invasive vagus nerve stimulation (nVNS): a proof-of-concept study. Brain Stimul. (2019) 12:643–51. 10.1016/j.brs.2019.01.008 30745260

